# The Drug Habit

**Published:** 1898-05

**Authors:** A. W. Harlan

**Affiliations:** Chicago, Ill.


ARTICLE III.

### THE DRUG HABIT.

A. W. HARLAN, M. D., D. D. S., CHICAGO, ILL.

Thirty or thirty-five years ago the custom of using washes, lotions and pastes in the mouth as a habit had not become firmly fixed, but after the introduction of the Listerian system of surgery a sort of epidemic of antisepticism struck the whole populace. Every one was seeking for an antiseptic to destroy some unseen, unknown, unfelt enemy to human health. It was believed, not only by the public, but by the practitioner, that it was an absolute necessity that every time a person awoke in the morning his or her mouth should be washed with something ending in *ine*, *ol*, or *at*, or some such ending as that, the sum and substance of the article used being derived from the name of the principal antiseptic. The habit of presribing washes and lotions and pastes for oral cleansing has been growing and has grown so enormously that to-day we are confronted with a possible condition of things which it may be difficult to eradicate. Our teeth and our mucous membranes, being bathed or sprayed once or twice or three times or oftener daily, have acquired what in general medicine would be called the drug habit, drug toleration, or drug necessity. The mucous membrane in the morning is dry or hot, not moist, not having a normal temperature; but soon, from the stimulating effects of the habitual antiseptic (?), the mouth assumes something of naturalness. The fact is that this custom of feeding the mucous membrane daily with sprays and washes and lotions and pastes, the composition of which is known to the taker, frequently unknown to the prescriber, is doing a vast amount of injury. Most of these so-called antiseptic lotions contain acids in varying degrees of strength. Acids in a high state of dilution, as most of you know, are more destructive to tissues like the teeth than those in a concentrated state, because those that are in a highly diluted state do not excite a quick flow of saliva to neutralize their effects. But those in a concentrated state cause such an outpouring of saliva and mucus that they are soon neutralized, or so changed, or they are thrown out of the mouth so quickly that no damage is done. It is the highly diluted, minimized acid that is so insidious, and its destructive influences are not seen or felt until the disaster has been accomplished. It is this habit of drug-taking and drug-using and continual drugging that we wish to protect against. There is no necessity for the use of the so-called antiseptic mouth washes and lotions and pastes in a state of health. It is only when the tissues of the mucous membrane of the mouth are abnormal, not performing their proper functions, that drugs should be used to correct any variation from the normal until Nature has a chance to reassert herself. If our forefathers had been supplied with listerine, pasteurine, borolyptol, borine, and sanitol, and numerous other preparations of this character, to-day probably we would not be blessed with the mucous membranes and the teeth that we have, because a long series of years, of generations of use of deleterious drugs must have had a modifying influence on the structures of posterity. We find with a state of normality of the mucous membranes and the teeth, that the use of warm water first with the mere act of cleansing, and the use of cold water immediately following for the refreshing shock that it produces, is all that is necessary. But when there are masses of putrefaction between the teeth and underneath the edges of the gum or gums, the mere introduction of a little spray of listerine, of borolyptol, or any of the other advertised mouth-washes will not destroy these masses. They must be gotten rid of in some other way, usually by some operative interference. What would be thought of a man who, when he got up in the morning, would rush to a bottle of boro-glycerine water and bathe his face in it, or a bottle of listerine, and bathe his face in it? Would he have that refreshing sense of ablution that one has from the use of cold water? Would his skin be bright? Would the blood rush to the surface? Is there any necessity for this continued, inefficient, useless antiseptic washing of the mucous membrane of the mouth with antiseptics, which are not even strong enough to destroy anything that would be dangerous, and which would only destroy innocent bacilli or micrococci, which would be destroyed mechanically without the use of such antiseptics?

The drug habit is not only confined to use in the mouth and around the teeth, but every time one has a little pain in the left side of the head, or all over it, then they want bromo-seltzer, bromo-soda, or bromo-caffeine. If they have a little cut or bruise, or something which Nature will take care of well, they must have a paste, a salve, or an antiseptic—something or other containing cocaine as one of the ingredients, to dress it with. The Spartan like principle which was so prevalent in the days of old seems to have departed from the present generation, and now we cannot have a little pain in the belly, the stomach, the toe, the head, or the ear, but what we must use some effervescing drug or other containing a deadly nervine which dulls the senses, dulls the appreciation of all the things in life that are worth living for, and later becomes fixed as a habit. It is within my own experience, working from day to day, that men and women spending so much time in the pursuit of idleness and frivolity are being kept alive, and what little vivacity they posess is due to the immoderate, unnecessary and continuous use of drugs.

I wish to say to you to-night, gentlemen, that the man who needlessly or causelessly fixes upon a patient the necessity for using the so-called feeble, unnecessary antiseptic washes, lotions, and pastes is doing great injury. Most of the erosions that we have on teeth, if not begun by the use of these feeble, diluted acid lotions and washes, are carried along by the injudicious and too frequent use of tooth-brushes, tooth rubbers, tooth-pastes and powders containing ingredients that are not soluble in the fluids of the mouth or in the water. Half of the ridges and grooves, and even disfigured faces of teeth that we see, are brought about through these means. What I would ask of you to do is to do as I have done—get a half-dozen or more samples of the washes and lotions and pastes that are sold, not by the direction of the physician or dentist, but sold by advertising through the daily press and elsewhere, and procurable at any drug store, any department store, or any place where such things can be sold, and containing the recommendations of dentists and physicians who have never examined them—I say, if you will get a few samples and test them, as I have done, you will find that nearly all of them have a decided acid reaction, which must be deleterious not only to the mucous membrane of the mouth, but to the teeth; and the greatest danger from the use of these is to the teeth first, but the secondary danger is to the mucous membrane, because the continued use of such drugs blunts the sensibility of the mucous membrane just as certainly and more destructively than the use of tobacco or chewing gums, or any other of the pernicious habits of the present civilization. What we want is scientific prescribing for abnormal conditions; not the indiscriminate use of the confectionery-tasting drugs for the prevention of some far-off ill which would be dangerous to the mucous membrane of the mouth, throat or teeth.

Preventive medicine rests on a sounder basis than the prescribing of nostrums or the ignorant use of them.

A growing evil is the too frequent use of chloroform to produce a partial anæsthesia for the relief of probable pain in the cutting of dentine. This is accomplished by inhalation. Its use is at first a pleasure to some and later it is used to alleviate imaginary or other ills, until it is relied upon to take the place of energy of mind or body or both. I regard the illegitimate use of chloroform in the practice of dental medicine or surgery as a crime against the community. It is only necessary to look at the statistics of death from its use of extractions alone to prove this. I protest against the induction of partial anæsthesia for the purpose mentioned, and also against it as a fascinating way of acquiring a drug habit. It needs no argument to prove that in the legitimate use of drugs many cases of the drug habit have been acquired, notably in the use of anodynes and soporifics in neuralgia, sciatica, and other affections of the nervous system. We are too prone to think that all of the little ills of life must be assuaged by the use of chloral, hemp, opium, cocaine, and coal tar extracts and others of the sedative class. A large regard for the future welfare of the race would lead us to look after ventilation of houses and school rooms, the use of water for bathing, the decrease of heat in our homes, the proper selection and cooking of nutritious food, well-aired and well-ventilated sleeping-rooms. Exercise and sleep are of so much importance that if we would have a sound mind in a sound body, both should be taught as a part of our common-school system. The rosy cheek and elastic footstep of youth would be seen and heard hourly by you if you would insist on the observance of a few plain precepts and examples and the dissemination of such accurate and pleasant rules as could easily be practiced with pleasure. Drugs would not fill every closet and shelf in every house where money can buy them, if people were told that necessity did not exist for their abuse or overuse. The drug toleration is no evidence of need; it is feeding a pernicious habit which grows through the laxness of laws, or the inefficient exercise of them; but more than all, the abuse grows out of the ignorance of the practitioner, who is loaded down with samples and requested to try them on his patients and friends. Woe to the public that is thus prescribed to, and upon which the intolerable tyranny of a drug habit is fixed!—*Dental Review*.

